# Integrated follicular fluid multi-omics identifies steroidogenic dysregulation and a candidate SHBG-associated rescue framework in poor ovarian response

**DOI:** 10.3389/fendo.2026.1909283

**Published:** 2026-09-01

**Authors:** Runzi Zheng, Yan Jiao, Jiapeng Liu, Wenxin Yang, Zhuoran Wang, Wenting Tang, Qijiao He, Ze Wu, Lifeng Xiang

**Affiliations:** 1 Faculty of Life Science and Technology, Kunming University of Science and Technology, Kunming, Yunnan, China; 2 The Affiliated Hospital of Kunming University of Science and Technology, Kunming, Yunnan, China; 3 Department of Reproductive Medicine, National Health Commission (NHC) Key Laboratory of Healthy Birth and Birth Defect Prevention in Western China, The First People’s Hospital of Yunnan Province, Kunming, Yunnan, China; 4 Kunming University of Science and Technology- Yunnan Province First People's Hospital (KUST-YPFPH) Reproductive Medicine Joint Research Center, Medical School of Kunming University of Science and Technology, Kunming, Yunnan, China; 5 Medical School, Kunming University of Science and Technology, Kunming, Yunnan, China

**Keywords:** follicular fluid, metabolomics, microbial signatures, multi-omics, poor ovarian response, SHBG, steroid biosynthesis

## Abstract

**Introduction:**

Poor ovarian response (POR) remains a major obstacle in assisted reproductive technology, yet the follicular microenvironmental determinants of impaired ovarian sensitivity are poorly understood. This study aimed to characterize the multi_omics landscape of the follicular fluid in POR and to explore potential therapeutic candidates and underlying mechanisms.

**Methods:**

We performed 16S rRNA sequencing and untargeted metabolomics on follicular fluid samples from 26 women with POR and 25 normoresponsive controls. Integrated cross_omics analysis, exploratory modelling, and age_adjusted sensitivity assessments were conducted. Candidate metabolites identified by MS/MS annotation were tested in a Tripterygium glycoside_induced ovarian injury mouse model, with subsequent ovarian RNA sequencing, molecular docking, molecular dynamics simulation, qPCR, and SHBG immunohistochemistry to interrogate downstream pathways.

**Results:**

POR was associated with reduced microbial diversity, 55 differential metabolic features, and convergence of 16S_based and metabolomic signals on ABC transporter_related pathways. Age_adjusted analyses indicated that the metabolomic component was more robust than the 16S community_level findings, which are interpreted as exploratory. Two downregulated metabolites --Harmalol and Beraprost --were prioritized for in vivo intervention. Both candidates partially restored follicle counts, reduced ovarian apoptosis, and improved LH/FSH profiles. Mechanistic investigations nominated an SHBG_associated steroidogenic program as a candidate downstream effector.

**Discussion:**

These findings support a working model wherein follicular microenvironment remodelling in POR converges on steroidogenic dysregulation, providing testable rescue hypotheses. The results highlight the relative robustness of metabolomic signatures over microbiome shifts in this context, though further functional validation is required to confirm causality and therapeutic potential.

## 1 Introduction

Poor ovarian response (POR) represents a major clinical challenge in reproductive medicine. It is primarily characterized by reduced ovarian sensitivity to exogenous gonadotropin stimulation, leading to fewer retrieved oocytes, higher cycle-cancellation rates, and poorer clinical pregnancy outcomes, thereby compromising the success of assisted reproductive technology (ART) procedures such as *in vitro* fertilization (IVF) (1, 2). Given the marked clinical heterogeneity of this patient population, the diagnosis and management of POR are still evolving. At present, the Bologna criteria proposed by the European Society of Human Reproduction and Embryology (ESHRE) remain widely used in clinical practice. According to these criteria, POR is diagnosed when at least two of the following conditions are present: advanced maternal age (≥40 years) or another known risk factor for POR, a previous episode of poor ovarian response (≤3 oocytes retrieved under a conventional stimulation protocol), and an abnormal ovarian reserve test such as reduced antral follicle count (AFC) or anti-Müllerian hormone (AMH) level (3). Simplified criteria based on prognostic indicators, including very low oocyte yield or AMH, have also been proposed to improve clinical operability (4). Epidemiological studies estimate that POR affects approximately 9-24% of women undergoing IVF, with incidence increasing with age, and its pathogenesis is influenced by a combination of genetic, immunological, environmental, and age-related factors (5–11).

Follicular fluid (FF) constitutes the immediate microenvironment of the developing oocyte and plays a pivotal role in determining oocyte quality and subsequent embryonic developmental potential (12). FF is a complex biological milieu composed of follicular-cell secretions and plasma exudates, containing hormones, growth factors, nutrients, and metabolites (13). This environment not only supplies energy substrates to the oocyte but also mediates bidirectional crosstalk between the oocyte and surrounding granulosa cells (14). Disturbance of FF composition may impair both nuclear and cytoplasmic maturation of oocytes and thereby influence downstream fertilization and embryogenesis (15). Accordingly, characterizing the molecular composition of FF provides an important opportunity to understand the pathological microenvironment associated with reproductive disorders.

In recent years, metabolomics has been widely applied to explore the mechanisms of reproductive pathologies by reflecting the dynamic alterations of pathological states (16). Previous studies have shown that altered FF metabolic profiles, particularly involving glucose, amino acid, and lipid metabolism, are associated with diminished ovarian function (17–20). Abnormal metabolite accumulation may disrupt local immunometabolic homeostasis in the ovary and adversely affect oocyte competence (21). In parallel, emerging evidence suggests that low-abundance microbial communities in FF may also influence oocyte development through metabolically active interactions (19). However, current POR research has largely focused on peripheral blood markers or single-omics metabolic screening of FF (22–24). As a result, the characteristics of the microbial component within the POR follicular microenvironment, and its crosstalk with the metabolome, remain insufficiently defined. This gap limits a deeper understanding of the pathological network underlying POR and weakens the search for mechanism-informed intervention strategies.

The shortage of mechanistic insight also constrains therapeutic development. Current management of POR mainly relies on individualized modification of ovarian stimulation protocols, nutritional supplementation such as antioxidants, and a range of adjuvant treatments. Yet protocol adjustment remains highly experience-dependent, while many nutritional and adjuvant approaches still lack strong evidence-based support, leading to uncertain efficacy and increased decision burden for patients (25–29). More advanced approaches, including platelet-rich plasma, stem cell therapy, and mitochondrial replacement, have shown promise but remain difficult to implement routinely because of their experimental status, lack of standardization, or ethical concerns (30–34). There is therefore a strong need to identify accessible and biologically grounded intervention targets from a molecular perspective.

Prompted by these considerations, we used an integrative multi-omics and computer-aided analysis strategy to investigate POR. We first characterized the microbial and metabolic features of FF in POR using 16S rRNA sequencing and untargeted metabolomics, and then explored their cross-omics relationships together with exploratory prediction modeling. Next, guided by MS/MS-annotated downregulated metabolic features, we used network pharmacology to prioritize Harmalol and Beraprost as candidate intervention compounds. In a mouse ovarian injury model, we evaluated their effects on follicular reserve and endocrine hormone profiles. Finally, through ovarian transcriptomic sequencing, molecular docking, molecular-dynamics simulation, qPCR validation, and SHBG immunohistochemistry, we examined whether these candidates might be associated with an SHBG-linked steroidogenic response. This study was designed to provide mechanistic hypotheses into microbiome-metabolome crosstalk in POR and to offer an experimental basis for future mechanism-guided intervention studies.

## 2 Materials and methods

### 2.1 Study participants

This case-control study was conducted in the Department of Reproductive Medicine, The First People’s Hospital of Yunnan Province, between January and March 2025. Participants were recruited among women undergoing *in vitro* fertilization (IVF) or intracytoplasmic sperm injection (ICSI). The inclusion criteria for all participants included an age range of 25 to 50 years. POR was diagnosed according to the Bologna criteria: (i) age >= 40 years or presence of risk factors; (ii) history of POR (<=3 oocytes retrieved after conventional stimulation); or (iii) diminished ovarian reserve, defined as antral follicle count (AFC) < 5–7 or anti-Mullerian hormone (AMH) < 0.5-1.1 ng/mL. After screening and obtaining informed consent, 26 women were enrolled in the POR group. For the revised analysis, sample-submission records matched to the omics sample identifiers were used to summarize available Bologna-criterion-related variables, including age >=40 years, diminished ovarian reserve, and index-cycle oocyte yield.

Exclusion criteria for the control group were: (i) age < 25 or > 35 years; (ii) any history of ovarian surgery (e.g., cystectomy, oophorectomy); (iii) conditions affecting ovarian reserve (e.g., endometriosis, polycystic ovary syndrome); and (iv) antibiotic treatment within the preceding three months. A total of 25 women meeting the criteria were enrolled in the control group. Thus, the final study sample comprised 51 women aged 25–50 years. This study was conducted in accordance with the Declaration of Helsinki and was approved by the Ethics Committee of The First People’s Hospital of Yunnan Province (Approval No. KHLL2024-KY310). Because the POR and control groups differed substantially in age distribution, age-adjusted sensitivity analyses were performed for the 16S and metabolomic datasets.

### 2.2 Sample collection

Ovarian stimulation protocols were individualized for each patient. Controlled ovarian hyperstimulation with exogenous gonadotropins (Gn) was initiated when LH < 5 U/L, E_2_ < 183.5 pmol/L, endometrial thickness < 4–5 mm, and no ovarian cysts were detected. In cases where cysts ≥ 3 cm were present, aspiration and pathological examination were performed before stimulation. Ultrasound and serum hormone monitoring were conducted from day 6 of stimulation. When 2–3 dominant follicles reached 18 mm, ≥ 60% of follicles were ≥ 15 mm, and serum E_2_ levels stabilized at 734–1101 pmol/L per mature follicle with rising P, serum LH, E_2_, P, and endometrial thickness (EMT) were recorded.

Ovulation trigger was determined by E_2_ levels: women with E_2_ <= 7340 pmol/L received Ovitrelle 250 ug plus HCG 2000 IU, those with E_2_ >= 18350 pmol/L received HCG 4000–5000 IU, and others received Ovitrelle 250 ug. Trigger was administered at a dose equivalent to 10 pmol to induce ovulation. Follicular fluid was collected from the first aspirated follicle during oocyte retrieval. Only clear follicular fluid without visible blood contamination was retained. Samples showing visible pink/red discoloration or gross blood contamination were excluded before aliquoting. Eligible follicular-fluid samples were divided into sterile 15 mL tubes, labeled, and stored at -80 degrees C until further analysis.

### 2.3 16S rRNA gene sequencing

Microbial DNA was extracted using the E.Z.N.A. Stool DNA Kit (Omega, USA). The V4 region of the 16S rRNA gene was amplified with primers 515F/806R, purified, quantified, and sequenced on the Illumina HiSeq platform (PE150). Because follicular fluid is a low-biomass specimen, negative controls were included in the 16S workflow. DNA extraction negative controls (D1-D5) and PCR negative controls (P1-P5) were processed together with study samples. Raw reads were filtered with fqtrim, merged with FLASH, and clustered into OTUs at 97% similarity using Vsearch. Taxonomy was assigned with the RDP classifier against the NT 16S database, and OTU tables were normalized to sequencing depth.

Alpha (Chao1, Observed species, Shannon, Simpson) and beta diversity were calculated in QIIME. Differential taxa were identified using Mann-Whitney U test, Kruskal-Wallis test, STAMP, and LEfSe. Functional prediction was performed with PICRUSt against KEGG pathways, and LEfSe was further applied to identify differentially enriched functions. Taxa identified as experimental contaminants from negative controls were flagged and removed from downstream analyses. The contaminant list included common low-biomass/reagent-associated genera, including Acinetobacter, Pseudomonas, Ralstonia, Burkholderia, Methylobacterium, Sphingomonas, and related taxa.

### 2.4 Metabolomics analysis

Metabolites were extracted from 100 μL samples with 80% methanol (1:4, v/v), centrifuged, dried, reconstituted in 80% methanol, and stored at −80 °C. QC samples were prepared by pooling equal aliquots. LC–MS was performed on an UltiMate 3000 UPLC (Waters ACQUITY UPLC T3 column, 100 × 2.1 mm, 1.8 μm) coupled to a Q-Exactive mass spectrometer (Thermo Scientific) in both ion modes. Data acquisition included full MS and data-dependent MS/MS. QC samples were injected every 10 runs.

Raw data were processed with XCMS, CAMERA, and metaX in R for peak detection, alignment, and annotation. Metabolites were annotated by accurate mass (<=10 ppm), isotopic distribution, and MS/MS spectra against KEGG, HMDB, and in-house libraries. Differential metabolic features were defined by fold change >1.2, p < 0.05 (t test), and VIP >1 from PLS-DA. KEGG pathway enrichment was performed using hypergeometric tests. Because metabolite identification was based on untargeted LC-MS/MS annotation without targeted analytical confirmation, candidate compounds were interpreted as MS/MS-annotated metabolic features rather than targeted-validated endogenous follicular-fluid metabolites.

### 2.5 Integrative functional analysis

Differential metabolites were identified based on the criteria of ratio ≥ 1.2 or ≤ 1/1.2, p value < 0.05, and VIP > 1. Differential microbial taxa were screened at the genus level with p < 0.05, and functional alterations of the microbiome were predicted using amplicon-based functional annotation, retaining significantly enriched KEGG terms. For metabolomics, significantly enriched KEGG pathways were selected from enrichment analysis results.

To explore shared functional alterations between the two datasets, differential KEGG pathways obtained from microbiome and metabolomics analyses were compared, and overlapping functional terms were identified using Venn analysis to define common functional signatures.

### 2.6 Integrative analysis

To evaluate the overall structural correlation between the microbiome and metabolome, a Mantel test was performed. The Bray-Curtis dissimilarity matrix for microbial taxa and the Euclidean distance matrix for metabolites were calculated, and the correlation between the two matrices was determined using the vegan package in R(v4.1.1).

For specific cross-omics interactions, the abundance data of differential microbial taxa and core metabolites were uploaded to the MetOrigin web platform (https://metorigin.met-bioinformatics.cn/home/). Spearman correlation analysis was conducted to construct microbe-metabolite interaction networks. Additionally, the integrated data were mapped to the Kyoto Encyclopedia of Genes and Genomes (KEGG) database via the platform’s built-in modules to perform functional enrichment analysis and identify jointly involved metabolic pathways.

### 2.7 Machine learning methods

To evaluate the exploratory classification potential of the identified signatures, three penalized regression algorithms--Ridge regression, Least Absolute Shrinkage and Selection Operator (Lasso) regression, and Elastic Net regression--were utilized to construct predictive models. The study cohort was randomly partitioned into a 70% training set and a 30% test set using the caret R package. To optimize model performance, mitigate overfitting, and handle potential multicollinearity among high-dimensional metabolic features, 10-fold cross-validation was performed to determine the optimal regularization parameters. The pROC package was employed to generate receiver operating characteristic (ROC) curves and calculate the area under the curve (AUC), which served as the primary metric to evaluate predictive performance across the training and test sets. Additional evaluative metrics, including accuracy, sensitivity, and specificity, were derived via confusion matrices. Because these models were developed in a single modest-sized cohort without external validation, they were interpreted as exploratory rather than clinically validated diagnostic models.

### 2.8 Network pharmacology and molecular docking

Drug structures were obtained from PubChem and imported into SwissTargetPrediction and SuperPred to predict potential targets. Predicted targets were merged and deduplicated. Disease-related targets for poor ovarian response were retrieved from GeneCards, OMIM, and DisGeNet using the keyword “poor ovarian response”, and intersected with drug targets to identify common genes.

Intersected targets were uploaded to STRING (v11.5; species: Homo sapiens) to construct protein–protein interaction (PPI) networks at a confidence threshold of 0.400. Networks were exported in PNG and TSV formats, and further analyzed in Cytoscape (v3.7.2) for topology and visualization. Functional enrichment analysis (GO: BP, CC, MF; KEGG pathways) was performed using the clusterProfiler package in R (v4.1.1), with results ranked by adjusted p-values (q-values). The top 20 terms were visualized with ggplot2.

Core hub proteins (high-degree nodes) were selected for molecular docking. Protein 3D structures (PDB format) were obtained from RCSB PDB and preprocessed with PyMOL (v4.6). Ligand 3D structures were downloaded from PubChem, converted from SDF to mol2 using OpenBabel (v2.4.1), and docked with protein targets in AutoDockTools (v1.5.6). Binding modes were visualized in PyMOL.

### 2.9 Animal model of POR and drug treatment

Fifty healthy 4-week-old female C57BL/6N mice were purchased from Charles River Laboratories (Beijing, China). Mice were randomly assigned to five groups (n = 10/group): Control, POR, M1 (beraprost), M2 (harmalol), and M3 (beraprost sodium). POR was induced in the POR, M1, M2, and M3 groups by daily oral gavage of Tripterygium glycosides suspension (QIANJIN, Z43020138, 50 mg/kg) for 2 weeks (35, 36). The control group received an equal volume of 0.9% NaCl. After modeling, mice in the M1 (0.6 mg/kg, HY-13569A, MCE) (37), M2 (67.2 mg/kg, HY-N2625A, MCE) (38), and M3 (0.6 mg/kg) groups received daily oral gavage for 1 week. At the end of treatment, mice were deeply anesthetized with isoflurane (4% for induction, 1.5% for maintenance) delivered in100% oxygen via a precision vaporizer. Anesthetic depth was confirmed by loss of pedal withdrawal reflex and corneal reflex. Blood was collected by terminal cardiac puncture using a 1-mL syringe with a 25G needle inserted at approximately 30–45°through the diaphragm, just below the xiphoid process, with gentle negative pressure. Following blood collection, mice were euthanized by cervical dislocation while still under deep anesthesia. Ovaries were then dissected, snap-frozen, and stored at −80 °C. All animal experiments were approved by the Institutional Animal Care and Use Committee (IACUC) of Committee of Kunming University of Science and Technology (Approval ID: AP-KUST-202412020031) and were conducted in accordance with relevant institutional and national guidelines. The study is reported in compliance with the Animal Research: Reporting of *In Vivo* Experiments (ARRIVE) guidelines.

### 2.10 Estrous cycle monitoring

Vaginal smears were collected daily by inserting a saline-moistened cotton swab (~0.4 cm) into the vaginal cavity, rotating gently, and smearing onto slides. Smears were stained with hematoxylin and eosin (HE), and estrous stages were determined under a light microscope based on epithelial cell morphology.

### 2.11 Serum hormone assays

Serum concentrations of AMH, FSH, LH, and E_2_ were determined using ELISA kits (AMH: Elabscience, E-EL-M3015; FSH: Elabscience, E-EL-M0511; LH: Reed Biotech, RE2741M; E_2_: Reed Biotech, RE10177) following the manufacturer’s instructions. Absorbance was read on a microplate reader, and concentrations were calculated from standard curves.

### 2.12 Biochemical analysis

Fresh serum or plasma samples were analyzed directly using an automated biochemical analyzer. Frozen samples were thawed on ice for 10–30 min before analysis, and samples with visible hemolysis were excluded. When sample volume was limited, assays were performed with appropriate dilution according to the instrument manual. During analysis, the analyzer automatically identified each sample through its barcode and carried out the entire workflow, including precise sample aspiration, reagent addition, mixing, incubation at 37°C, and photometric measurement at specific wavelengths. Absorbance was determined either at the reaction end point or continuously to calculate the rate of change.

### 2.13 Hematoxylin-eosin staining

Ovaries were fixed in 4% paraformaldehyde for 48 h, dehydrated, embedded in paraffin, and sectioned (5 μm). Sections were stained with hematoxylin (3–5 min) and eosin (5 min), dehydrated through graded ethanol, and mounted. Follicular development and atresia were evaluated under light microscopy.

### 2.14 TUNEL (Terminal Deoxynucleotidyl Transferase mediated dUTP Nick-End Labeling) assay

Ovarian apoptosis was assessed by TUNEL staining on paraffin sections. After deparaffinization and antigen retrieval, sections were incubated with TUNEL reaction mixture, counterstained with DAPI, and imaged under a fluorescence microscope. Apoptotic index was calculated as apoptotic nuclei/total nuclei × 100%.

### 2.15 RNA extraction, sequencing and data analysis

Total RNA was extracted using TRIzol reagent (Invitrogen, USA), and RNA quality was assessed with a NanoDrop 2000 and Agilent 2100 Bioanalyzer. Libraries were constructed with the VAHTS Universal V10 RNA-seq Library Prep Kit and sequenced on the Illumina NovaSeq 6000 platform (OE Biotech, Shanghai, China). Raw reads were filtered using fastp, mapped to the reference genome with HISAT2, and quantified with HTSeq-count. Gene expression levels were calculated as FPKM. Differentially expressed genes (DEGs) were identified with DESeq2 (q < 0.05, |fold change| > 1). PCA, clustering, and radar plots were performed in R. GO, KEGG, and GSEA were conducted using clusterProfiler, with hypergeometric testing for over-representation and adjusted p-value < 0.05 as the cutoff. GSEA was applied to the ranked gene list to detect coordinated pathway changes, following workflows commonly used in network pharmacology. FASTQ files and expression matrices from RNA-seq data are available from the National Library of Medicine database under the accession number: BioProject: PRJNA1335391.

### 2.16 Molecular dynamics simulation

MD simulations of 100 ns were performed using GROMACS 2023.2. The CHARMM36 all-atom force field was employed to construct the protein topology, whereas ligand parameters were generated using the General AMBER Force Field (GAFF) via Sobtop. The protein-ligand complex was placed in a cubic box with a minimum distance of 1.0 nm between the complex and the box boundaries. The system was solvated using the CHARMM-modified TIP3P water model. To maintain system neutrality and simulate a physiological environment, sodium and chloride ions (Na^+^ and Cl^-^) were added to reach a salt concentration of 0.15 M.

Initial steric clashes were removed through energy minimization using the steepest descent algorithm. Subsequently, the system underwent equilibration under the NPT ensemble with position restraints applied to the ligand. The final production MD simulation was executed for 100 ns. Trajectory analyses, including Root Mean Square Deviation (RMSD), Root Mean Square Fluctuation (RMSF), radius of gyration (Rg), number of intermolecular hydrogen bonds, and Solvent Accessible Surface Area (SASA), were calculated using GROMACS built-in toolkits. Additionally, the Free Energy Landscape (FEL) was constructed using the gmx sham module to map the lowest-energy conformational states. Data visualization and plotting were performed using DuIvyTools.

### 2.17 Quantitative PCR

Total RNA was isolated from tissues and blood using the RNeasy Mini Kit (Qiagen) or TRIzol reagent. Reverse transcription was performed with HiScript III RT SuperMix (Vazyme, 7110203). qPCR was conducted using a Roche LightCycler 96 with GAPDH as the internal control. Reactions were performed in duplicate, and relative gene expression was calculated using the 2^-ΔΔCt method. Primer sequences are listed in **Supplementary Table 3**.

### 2.18 Immunohistochemistry

Paraffin-embedded ovarian sections were deparaffinized, subjected to antigen retrieval, and blocked with 5% BSA (Beyotime, C0265). Sections were incubated overnight with primary antibody against SHBG (Proteintech, 18200-1-AP, 1:500), followed by HRP-conjugated secondary antibody (Beyotime, A0208, 1:50). Signals were developed with DAB and counterstained with hematoxylin. Images were acquired under light microscopy, and mean optical density was quantified across random fields.

### 2.19 Statistical analysis

All data are expressed as mean ± SEM. Statistical analyses were performed using GraphPad Prism v8. Comparisons between two groups were conducted using two-tailed unpaired Student’s t-test (Welch’s correction applied when variance was unequal). One-way ANOVA followed by Tukey’s or Dunnett’s *post hoc* test was used for multiple-group comparisons. A p-value < 0.05 was considered statistically significant.

## 3 Results

### 3.1 Follicular fluid metabolomics distinguishes POR from controls

**Figure 1:**
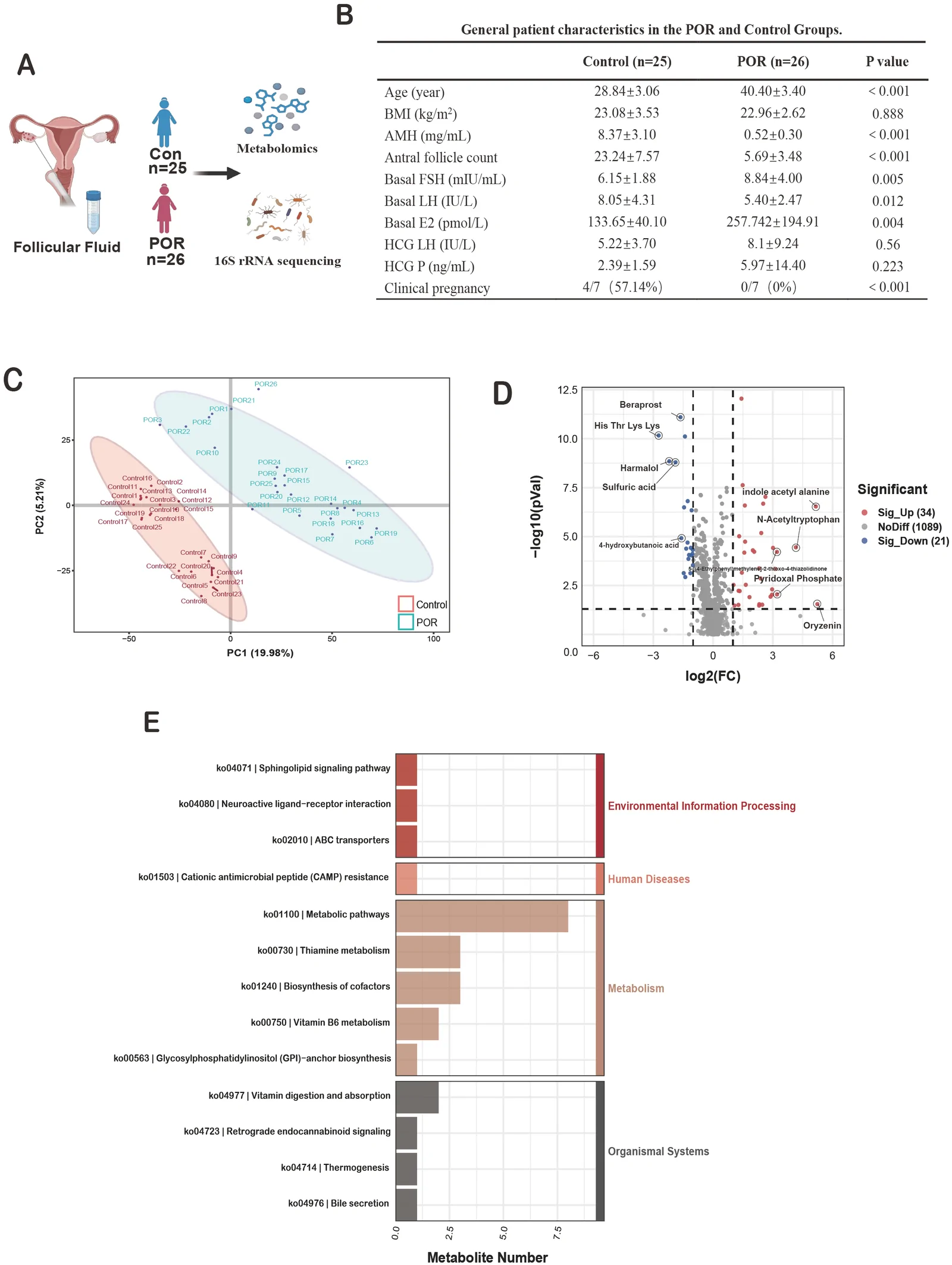
Metabolomic alterations in follicular fluid of patients. **(A)** Schematic representation of the experimental design for the POR and control groups. **(B)** General patient characteristics in the POR and control groups. **(C)** PCA of metabolites in the control and POR groups. **(D)** Volcano plot of differentially abundant metabolites between the POR and control groups. **(E)** KEGG pathway analysis of the differentially abundant metabolites. PCA, principal component analysis.

The overall study design is summarized in **Figure 1A**. We analyzed follicular fluid from 26 women with POR and 25 controls. As shown in **Figure 1B**, the POR cohort displayed the expected clinical phenotype of impaired ovarian reserve, including lower AMH and AFC values and altered basal gonadotropin and estradiol profiles; the POR group was also older than the control group. The control group had a mean age of 28.84 +/- 3.06 years, whereas the POR group had a mean age of 40.49 +/- 3.41 years (P = 2.86e-17; **Supplementary Table 5**). Within the POR cohort, available Bologna-criterion-related variables showed that 14 of 26 participants were >=40 years old, all 26 met the diminished ovarian reserve criterion, and all 26 had <=3 oocytes retrieved in the index cycle (**Supplementary Table 4**). At the global metabolomic level, principal component analysis separated POR samples from controls (**Figure 1C**). Exploratory within-POR subgroup analyses comparing patients with age >=40 years plus diminished ovarian reserve (n = 14) with younger patients with diminished ovarian reserve (n = 12) did not show robust subtype-driven separation. Specifically, no alpha-diversity metric remained significant after BH correction, Bray-Curtis beta diversity did not separate the two POR subgroups (pseudo-F = 0.562, P = 0.741), and no metabolomic feature remained significant after BH-FDR correction (**Supplementary Tables 10–S12**).

To place the differential-metabolite screen in a wider metabolic context, we first examined the overall annotation landscape. Broad KEGG annotation showed that the detected metabolic features were concentrated in general metabolic pathways and glycerophospholipid metabolism, supporting lipid remodeling as a prominent component of the POR follicular milieu (**Supplementary Figure 1A**). Differential analysis then identified 55 altered metabolites, including 34 increased and 21 decreased features in POR (**Figure 1D**). Sample-level abundance patterns of these metabolites were consistent across the cohort and are shown in the accompanying heatmap (**Supplementary Figure 1B**). KEGG enrichment of the differential metabolite set further highlighted global metabolic pathways, glycerophospholipid metabolism, vitamin-related pathways, and ABC transporter-related terms (**Figure 1E**). Together, these results indicate that POR is associated with substantial disturbance in FF metabolic composition, with a prominent signal involving lipid handling and metabolite-transport-associated functions.

Age-adjusted sensitivity analyses showed that the 16S community-level findings were sensitive to age adjustment. For alpha diversity, the group association for Shannon diversity was nominal but did not remain significant after BH correction (**Supplementary Table 6**). For Bray-Curtis beta diversity, an age-conditioned permutation analysis did not identify a significant group effect (**Supplementary Table 7**). Age-adjusted key-taxon models are provided in **Supplementary Table 8**. In contrast, the metabolomic differences showed greater consistency after age adjustment. Among the 771 features identified as differential in the primary analysis, 763 (99.0%) retained the same direction of effect in the age-adjusted model, 524 retained nominal group significance (P < 0.05), and 165 remained significant after BH-FDR correction (BH-FDR < 0.05). Across all 9733 detected metabolomic features, 317 features reached age-adjusted BH-FDR < 0.05 (**Supplementary Table 9**). These analyses support a more robust metabolomic difference between POR and controls, whereas the microbiome findings should be interpreted as exploratory.

### 3.2 16S-based microbial profiles show reduced diversity and Gammaproteobacteria enrichment in POR

**Figure 2:**
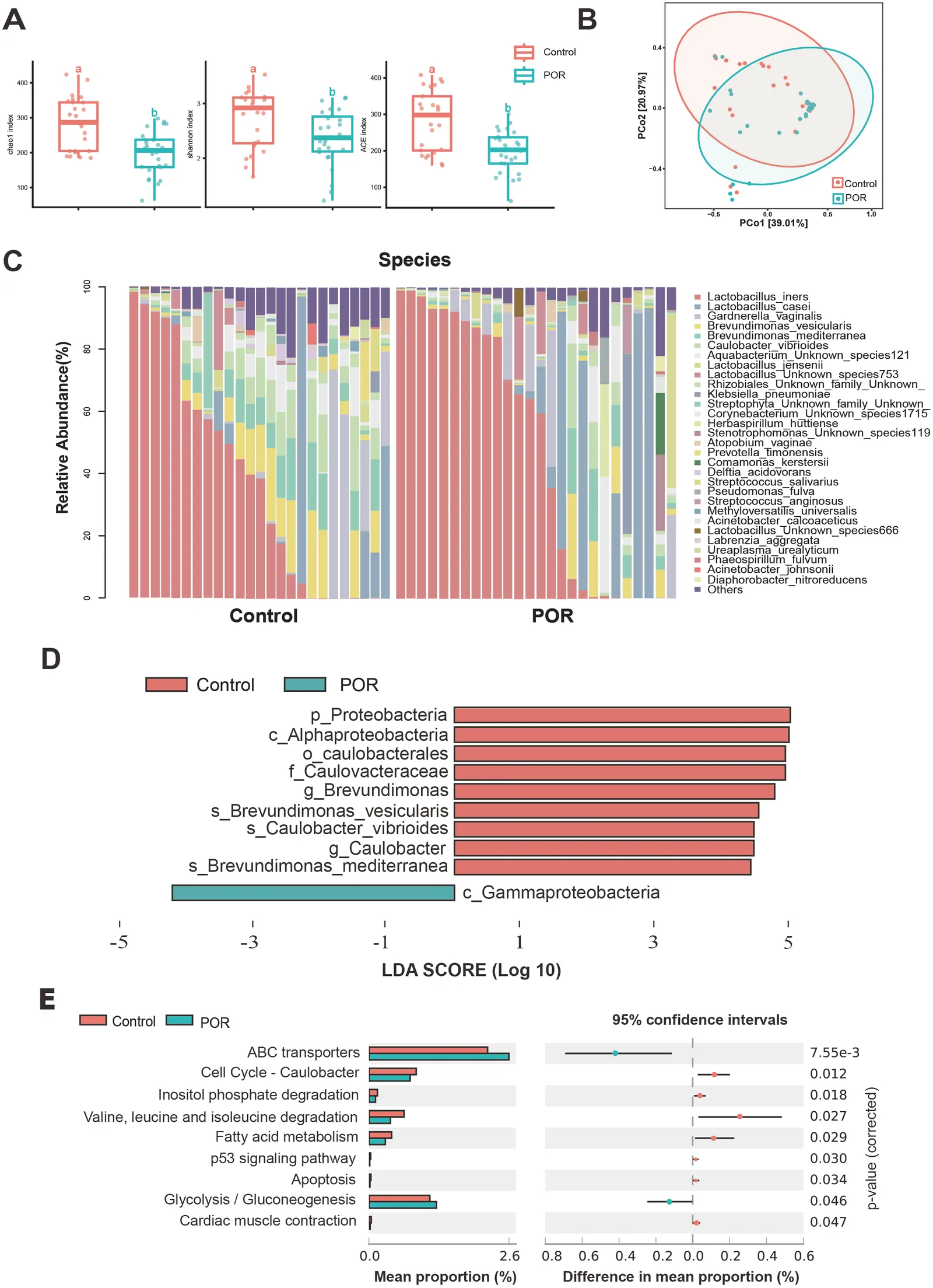
Alterations in the microbial communities of follicular fluid associated with patients with POR. **(A)** Alpha diversity indices (Chao1, Shannon, and ACE) of the microbiota in the control and POR groups. **(B)** Beta-diversity analysis using principal coordinate analysis (PCoA). **(C)** The relative abundance of microorganisms at the species level in the two groups. **(D)** LEfSe analysis identifying differentially abundant microbial taxa with LDA scores. **(E)** Predicted functional pathway differences of the microbiota between the control and POR groups. LDA, linear discriminant analysis.

16S rRNA analysis showed reduced alpha diversity in POR across the Chao1, Shannon, and ACE indices (**Figure 2A**), together with a distinct separation of beta diversity by principal coordinate analysis (**Figure 2B**). Lactobacillus remained the dominant genus in both groups (**Figure 2C**), but differential-taxon analysis identified a shift in the relative structure of the low-abundance community.

In particular, Gammaproteobacteria-related signatures were enriched in the POR group, whereas Alphaproteobacteria and related taxa, including Brevundimonas and Caulobacter, were more strongly represented in controls (**Figure 2D**). Functional prediction of the 16S dataset suggested increased ABC transporter and glycolysis/gluconeogenesis pathway signals in POR, accompanied by reduced branched-chain amino acid degradation and fatty acid metabolism (**Figure 2E**). Although these inferred functions require direct validation and may be affected by the low-biomass nature of follicular fluid, they align with the metabolic remodeling observed above and support the presence of disturbed energy and lipid homeostasis in the POR follicular environment.

### 3.3 Cross-omics integration highlights ABC transporter-related pathway overlap and exploratory prediction signatures

**Figure 3:**
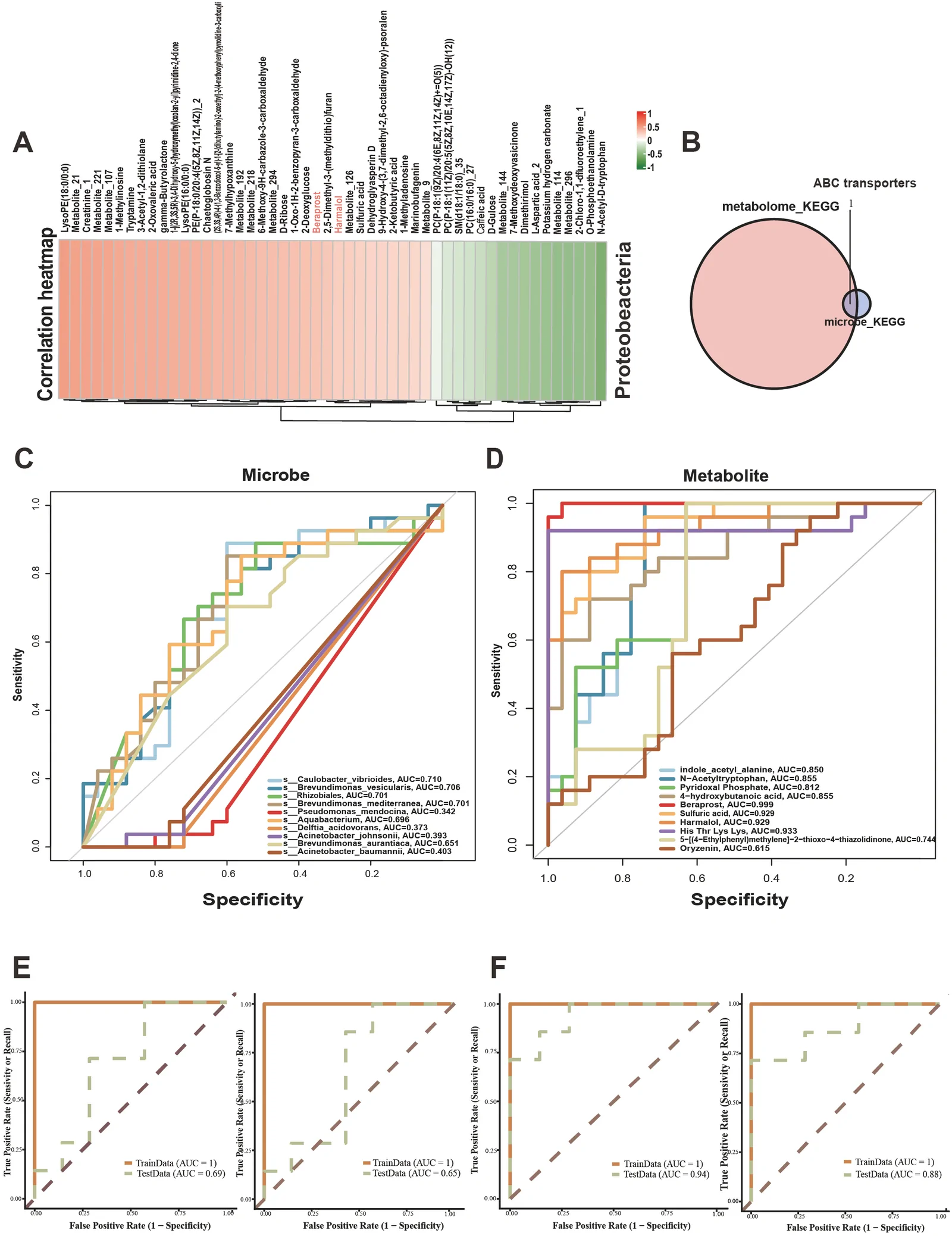
Integrative analysis of the microbiome and metabolome and machine learning-based diagnostic models. **(A)** Correlation heatmap of the relative abundance of Proteobacteria and differential metabolites. **(B)** Venn diagram illustrating the intersection of KEGG pathways enriched by both differential microorganisms and metabolites. **(C)** Receiver operating characteristic (ROC) curves for individual differential microorganisms. **(D)** ROC curves for individual differential metabolites. **(E)** ROC curves evaluating the predictive performance of Elastic Net (left) and Ridge regression models (right) based on differential microorganisms. **(F)** ROC curves evaluating the predictive performance of Elastic Net (left) and Ridge regression models (right) based on differential metabolites. ROC, Receiver operating characteristic.

To define the relationship between FF microbial profiles and metabolites, we next performed cross-omics integration analyses. At the global level, the Mantel-test network supported an overall association between microbial and metabolic distance structures (**Supplementary Figure 1C**). At finer resolution, Proteobacteria-related taxa, including Acinetobacter johnsonii, Delftia acidovorans, and Pseudomonas mendocina, showed significant associations with lipid-derived metabolites and steroid-related intermediates (**Figure 3A**), supporting the presence of coordinated microbial-metabolic remodeling within FF.

When enrichment results from the microbial and metabolomic datasets were intersected, ABC transporter-related pathways emerged as the only shared functional term (**Figure 3B**). This result does not by itself establish transporter dysfunction, but it identifies transporter-associated biology as a reproducible convergence point across the two omics layers (39, 40). We then assessed the classification potential of the resulting signatures. Individual microorganisms and metabolites each showed baseline discriminatory performance (**Figures 3C, D**), and multivariable exploratory models based on microbial features yielded test-set AUCs of 0.69 and 0.65 for Elastic Net and Ridge regression, respectively (**Figure 3E**). Metabolite-based models performed better, with test-set AUCs of 0.94 and 0.88 (**Figure 3F**). Given the modest cohort size, we interpret these models as exploratory rather than clinically validated.

### 3.4 Harmalol- and beraprost-based interventions partially rescue ovarian injury *in vivo*

Among the MS/MS-annotated downregulated metabolic features observed in POR, Harmalol and Beraprost were prioritized for downstream testing on the basis of annotation confidence, ranking, literature support, chemical accessibility, and network-pharmacology follow-up (41–45).

**Figure 4:**
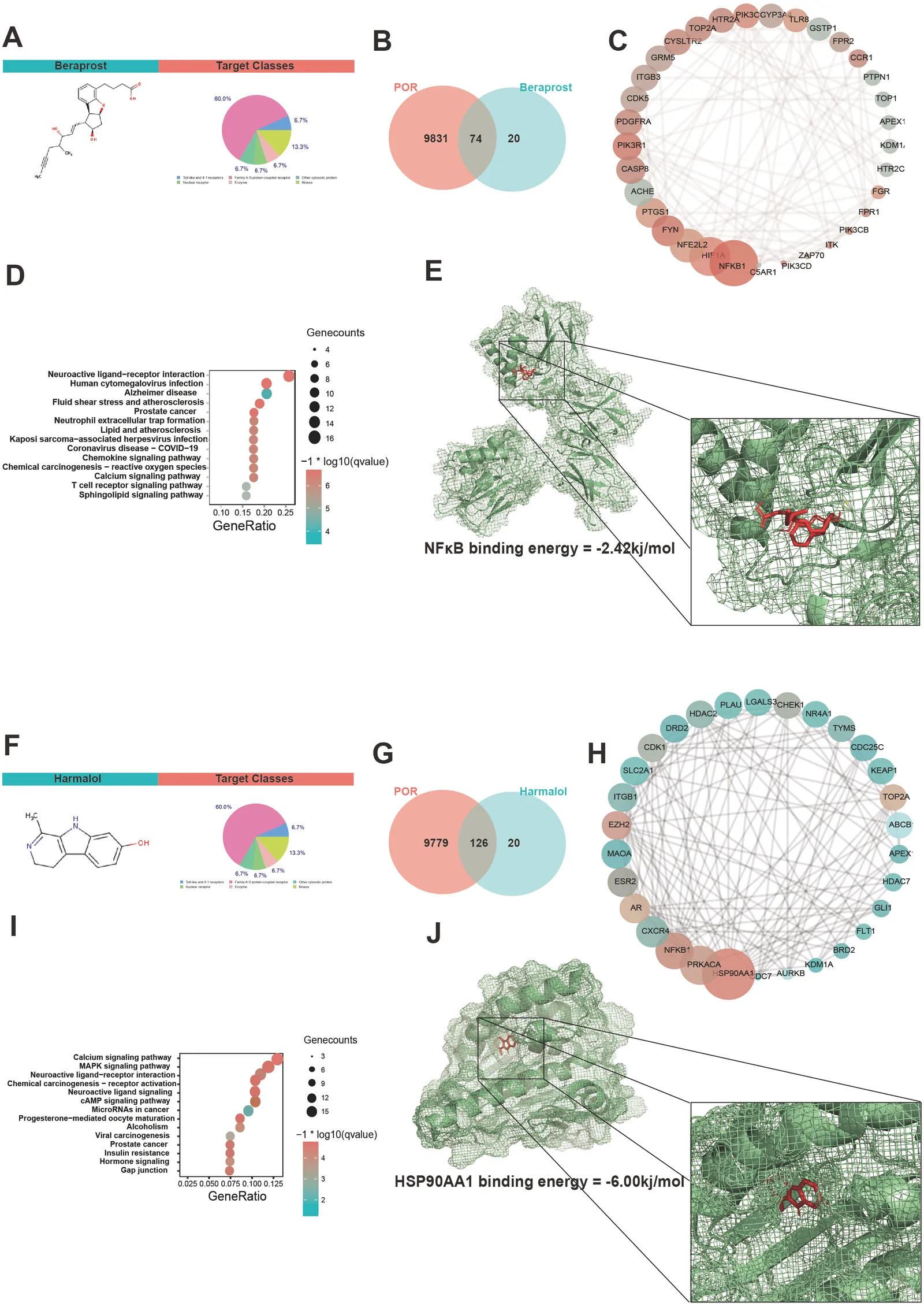
Network pharmacology analysis and molecular docking of the candidate metabolites Beraprost and Harmalol. **(A)** Chemical structure and target class distribution of Beraprost. **(B)** Venn diagram illustrating the overlapping targets between POR and Beraprost. **(C)** PPI network of the intersecting targets for Beraprost. **(D)** KEGG pathway enrichment analysis of the overlapping targets for Beraprost. **(E)** Molecular docking model and binding energy of Beraprost with NF-κB. **(F)** Chemical structure and target class distribution of Harmalol. **(G)** Venn diagram illustrating the overlapping targets between POR and Harmalol. **(H)** PPI network of the intersecting targets for Harmalol. **(I)** KEGG pathway enrichment analysis of the overlapping targets for Harmalol. **(J)** Molecular docking model and binding energy of Harmalol with HSP90AA1. PPI, Protein-protein interaction.

Beraprost was represented by feature NEG_397.205_3.6184 (MS2 score 70.3; FC = 0.3224; original q = 6.998e-09; age-adjusted group P = 0.00119; BH q = 0.0443). Harmalol was represented by feature NEG_199.093_5.1468 (MS2 score 95.34; FC = 0.2181; original q = 4.426e-07; age-adjusted group P = 0.00285; BH q = 0.0653). Their chemical structures, target-distribution summaries, and disease-target intersection analyses are shown in **Figures 4A, B, F, G**. Network-pharmacology analysis predicted 94 putative targets for Beraprost and 146 for Harmalol, and intersection with POR-related gene sets identified 74 and 146 overlapping disease-associated targets, respectively. Protein-network and pathway-enrichment analyses linked these targets to signaling processes that included calcium signaling, kinase activity, neuroactive ligand-receptor interaction, and MAPK-related biology (**Figures 4C, D, H, I**). The corresponding GO-level enrichment patterns are provided as supplementary support (**Supplementary Figures 2A, B**).

**Figure 5:**
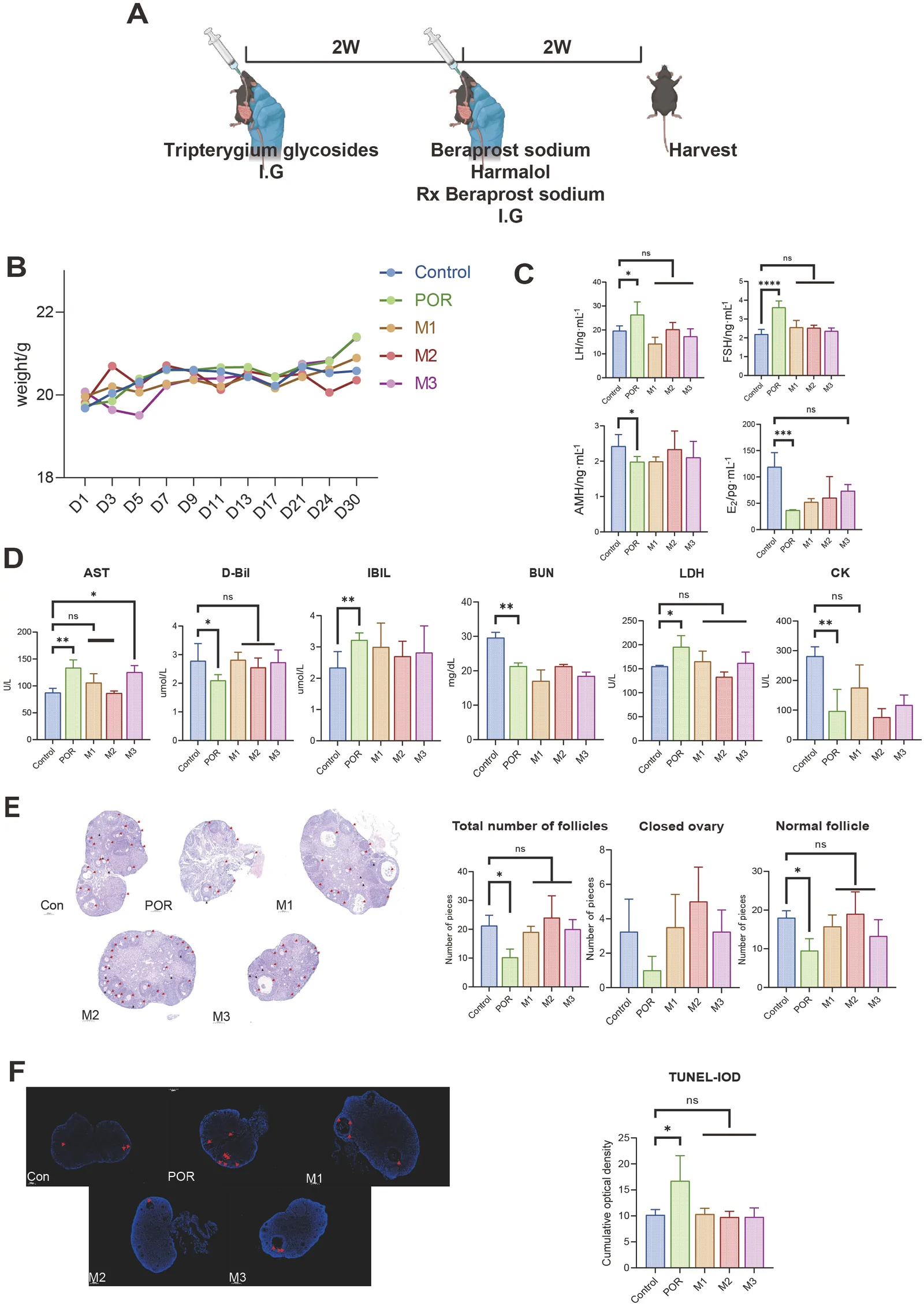
*In vivo* evaluation of the therapeutic effects of candidate interventions in the POR mouse model. **(A)** Schematic representation of the *in vivo* experimental design. **(B)** Body weight changes of mice across different groups during the intervention period. **(C)** Serum reproductive endocrine hormone levels determined by ELISA. **(D)** Analysis of blood biochemical parameters. **(E)** Representative images of H&E-stained ovarian sections and quantification of follicle numbers. **(F)** Representative images of TUNEL-stained ovarian sections and quantification of apoptotic cells.

Docking suggested favorable binding conformations for Harmalol to several hub proteins, whereas Beraprost showed weaker binding to NF-kB but sufficient translational relevance to justify *in vivo* follow-up because of its clinical accessibility (**Figures 4E, J**). Additional docking views for representative complexes are shown in **Supplementary Figures 2C, D** (46, 47). We therefore evaluated beraprost- and Harmalol-based interventions in a Tripterygium glycoside-induced ovarian injury model, as outlined in **Figure 5A**.

No major treatment-related body-weight changes were observed during the intervention period (**Figure 5B**). In the model group, serum LH and FSH were elevated and AMH and estradiol were reduced, confirming endocrine disruption; treatment suppressed the abnormal LH and FSH increase toward control levels, whereas AMH and estradiol showed partial upward trends (**Figure 5C**). Biochemical profiling did not indicate overt systemic toxicity and suggested partial improvement of stress-related serum abnormalities (**Figure 5D**). Continuous vaginal smear monitoring provided additional support that the interventions did not introduce obvious extra disruption of the estrous cycle (**Supplementary Figure 3A**).

Histological analysis further showed depletion of the follicular reserve in the model group, with decreases in total and morphologically normal follicles (**Figure 5E**). All three intervention arms increased follicle counts relative to untreated model animals, although atretic follicles also rose in parallel, indicating incomplete structural rescue. TUNEL staining demonstrated reduced ovarian apoptosis after treatment (**Figure 5F**). Together, these data support partial phenotypic rescue of ovarian injury by Harmalol- and beraprost-based interventions.

### 3.5 Ovarian transcriptomics and SHBG immunohistochemistry nominate a candidate steroidogenic rescue program

**Figure 6:**
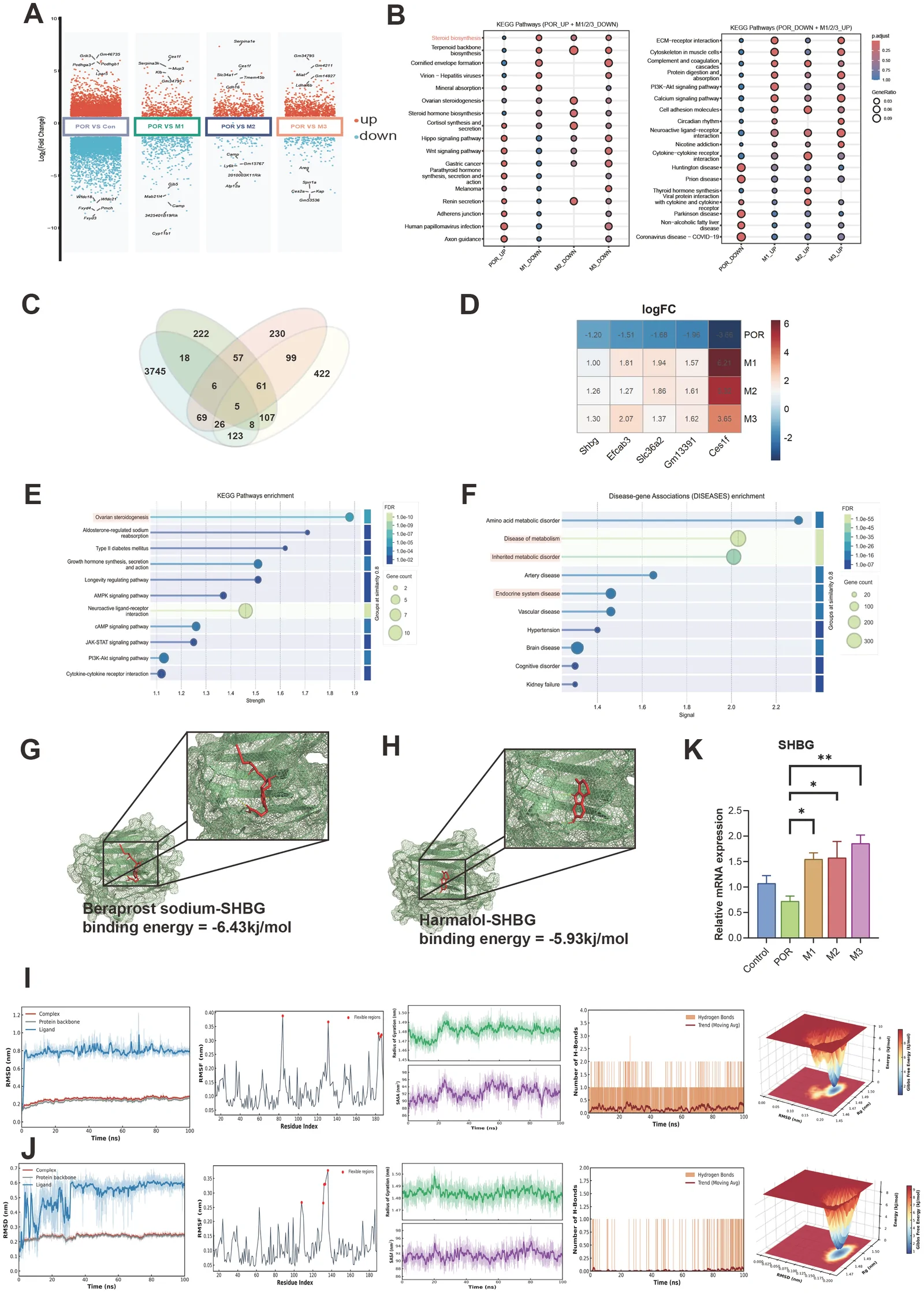
Identification of SHBG as the core target and structural dynamics validation of candidate interventions. **(A)** Volcano plots of differentially expressed genes (DEGs) in each comparison group. **(B)** Clustered bubble chart showing the intersecting KEGG pathways downregulated in the POR group and upregulated by the intervention drugs. **(C)** Venn diagram illustrating the intersection of DEGs among the POR and intervention groups. **(D)** Heatmap displaying the logFC values of the five intersecting core genes across different groups. **(E)** KEGG pathway enrichment analysis of the SHBG-associated PPI. **(F)** Disease-gene associations enrichment analysis of the SHBG-associated network. **(G, H)** Molecular docking models and binding energies of Beraprost sodium **(E)** and Harmalol **(F)** with SHBG. **(I, J)** 100-ns molecular dynamics simulation trajectories, including RMSD, RMSF, Rg, SASA, number of hydrogen bonds, and FEL, for the Beraprost sodium-SHBG **(I)** and Harmalol-SHBG **(J)** complexes. **(K)** Relative mRNA expression of Shbg in mouse ovarian tissues detected by RT-qPCR. RMSD, Root Mean Square Deviation. RMSF, Root Mean Square Fluctuation. Rg, Radius of Gyration. SASA, Solvent Accessible Surface Area. FEL, free energy landscape.

To identify downstream mechanisms linked to the observed *in vivo* rescue, we performed ovarian RNA sequencing. Volcano plots summarizing the differential transcriptional responses are shown in **Figure 6A**. Relative to controls, the model group showed 2550 differentially expressed genes, including 914 upregulated and 1636 downregulated transcripts. Each intervention partially reshaped the ovarian transcriptome, with 553 upregulated and 72 downregulated genes in the M1 arm, 851 upregulated and 202 downregulated genes in the M2 arm, and 484 upregulated and 400 downregulated genes in the M3 arm. At the pathway level, complementary KEGG summaries for the POR, M1, M2, and M3 transcriptional response patterns are provided in **Supplementary Figure 3B**, while GO enrichment of downregulated and upregulated gene sets across groups is shown in **Supplementary Figure 2**.

Cross-comparison of pathway changes across treatment groups highlighted steroid biosynthesis, terpenoid backbone biosynthesis, and ovarian steroidogenesis as consistently responsive pathways (**Figure 6B**). Gene set enrichment analysis further supported the repeated downregulation of the steroid biosynthesis program after treatment relative to the injury model, suggesting that normalization of steroidogenic dysregulation is a common response shared by the candidate interventions. The corresponding GSEA results are shown in **Supplementary Figure 3C**.

**Figure 7:**
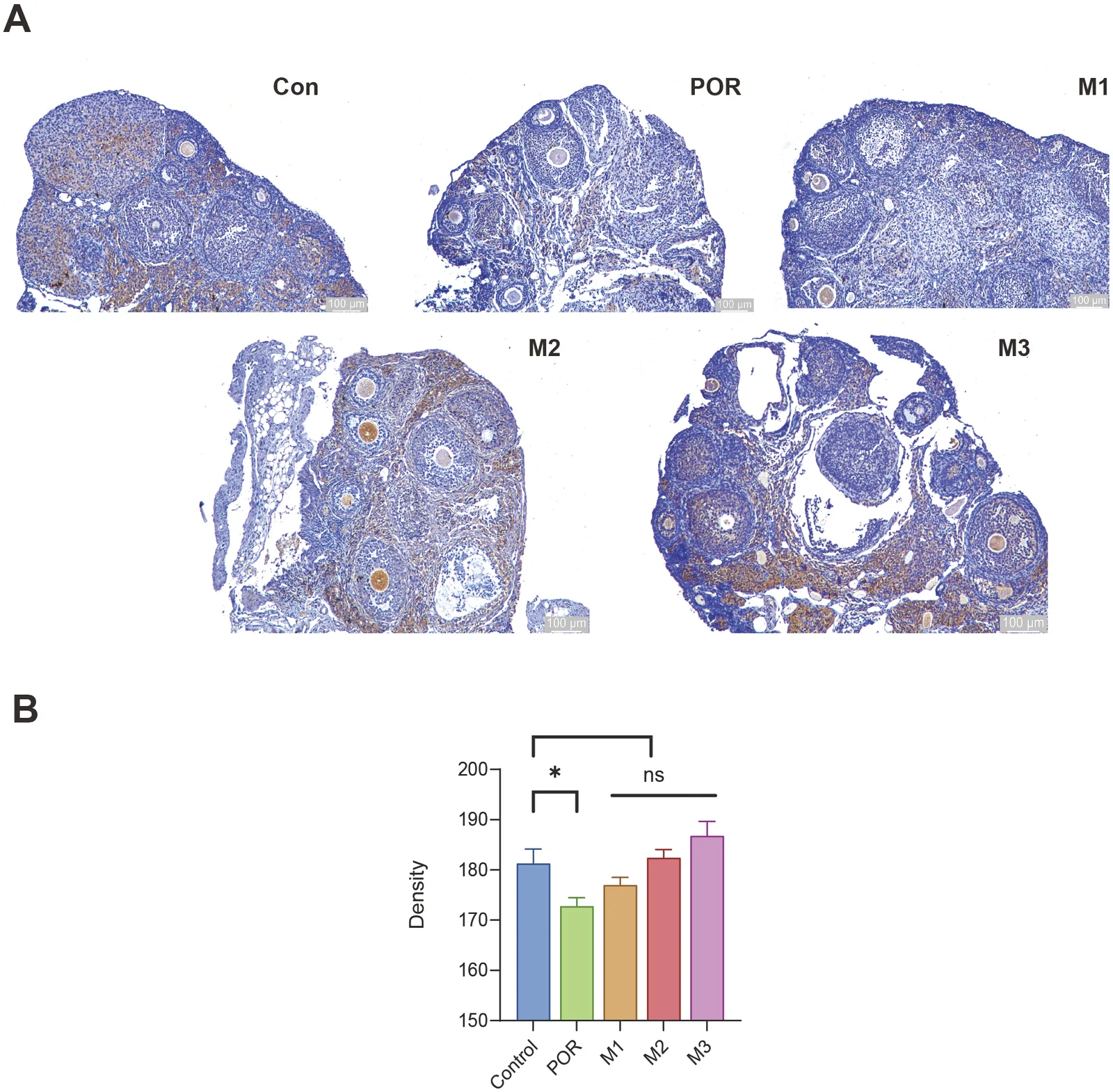
Immunohistochemical analysis of SHBG in mouse ovaries. **(A)** Representative SHBG staining in ovaries from each group. **(B)** Quantification of SHBG immunostaining.

We next intersected genes that were reduced in the model group but restored by all three interventions and identified five overlapping candidates: Shbg, Efcab3, Slc36a2, Gm13391, and Ces1f (**Figures 6C, D**). We prioritized Shbg because of its known endocrine relevance and its connection to steroid-hormone homeostasis. Protein-network and disease-ontology analyses linked SHBG-associated genes to ovarian steroidogenesis and reproductive or endocrine disease categories (**Figures 6E, F**). Docking and 100 ns molecular-dynamics simulations supported stable binding of beraprost sodium and Harmalol to SHBG, with binding energies of -6.43 kJ/mol and -5.93 kJ/mol, respectively (**Figures 6G–J**). Ovarian qPCR confirmed that Shbg expression was reduced in the injury model and restored by all intervention arms (**Figure 6K**). To provide protein-level and tissue-localization support, we added SHBG immunohistochemistry in ovarian sections. SHBG staining was reduced in the ovarian injury model compared with controls and showed partial recovery after candidate-compound treatment (**Figures 7A, B**). Collectively, these findings nominate an SHBG-associated steroidogenic rescue axis as a candidate downstream mechanism, while functional perturbation experiments remain necessary to establish causality.

## 4 Discussion

The pathogenesis of POR is highly complex, and mechanism-based standard interventions remain limited in current clinical practice. Previous studies have mainly focused on identifying metabolic biomarkers in peripheral blood or follicular fluid from POR patients (23, 48), with much less attention given to upstream drivers and translational intervention strategies. In the present study, we integrated 16S rRNA sequencing with untargeted metabolomics to characterize a coordinated microbial-metabolic remodeling pattern in FF. More importantly, we extended the work beyond descriptive multi-omics profiling by prioritizing candidate intervention compounds from MS/MS-annotated downregulated metabolic features and testing them *in vivo*. In this sense, the study attempts to connect molecular discovery with pharmacologic follow-up within a single experimental framework.

Dysbiosis of the reproductive tract microbiome has been linked to adverse reproductive outcomes (49). In our data, the POR-associated FF profile was characterized by reduced microbial diversity together with enrichment of Gammaproteobacteria-related signatures and marked perturbation of local lipid and energy metabolism. Cross-omics correlation analysis further showed strong coupling between microbial shifts and metabolic disturbance, while pathway intersection highlighted ABC transporter-related biology as the principal shared signal across the two datasets. ABC transporters, including members such as ABCA1 and ABCG2, mediate ATP-dependent transport of lipids, sterols, and other metabolites and are central to intracellular homeostasis (50, 51). These observations support the possibility that microbial succession and metabolic imbalance in POR may converge on transport-related dysfunction within the follicular environment. At the same time, this point should be stated with caution: our evidence supports pathway-level convergence rather than direct demonstration of transporter impairment.

The transporter-associated interpretation is relevant because steroidogenesis in follicular cells depends on the coordinated movement and conversion of sterol precursors. In our intervention experiments, steroid biosynthesis and ovarian steroidogenesis emerged as the most consistent treatment-responsive pathways in the ovarian transcriptome, and the supplementary KEGG and GSEA analyses reinforced this pattern. It is therefore plausible that the disturbed follicular microenvironment in POR contributes to impaired steroidogenic organization, which may in turn affect endocrine feedback and follicular development. Rather than claiming a fully resolved causal chain, our data support a working model in which microbial-metabolic remodeling, transport-associated pathway disturbance, and steroidogenic dysregulation are biologically connected layers of the same pathological process.

To translate these findings toward intervention, we focused on MS/MS-annotated downregulated metabolic features and prioritized Harmalol and Beraprost through network pharmacology and structural screening. Both compounds were linked to the altered cross-omics landscape and subsequently showed partial rescue effects in the Tripterygium glycoside-induced ovarian injury model, including improvement in endocrine readouts, follicle counts, and apoptosis. Beraprost is a clinically used prostacyclin analogue with anti-inflammatory and microcirculation-improving properties (39, 40, 46), whereas Harmalol is a natural product with documented bioactivity (41–43).These observations suggest that the altered FF landscape may contain chemically actionable signals. However, the current results do not prove that the human FF annotations represent definitively validated endogenous metabolites, nor do they establish that the *in vivo* rescue effects operate through exactly the same route suggested by the discovery-layer analyses. The intervention data should therefore be viewed as supportive translational evidence rather than final mechanistic confirmation.

Among the downstream signals, SHBG emerged as the most interesting candidate node. SHBG is a major regulator of circulating free sex hormones, and low SHBG levels have been associated with reproductive endocrine disorders such as polycystic ovary syndrome (47). In our dataset, Shbg was one of only a few genes consistently restored across all intervention groups, and the associated network was enriched for ovarian steroidogenesis and reproductive or endocrine disease categories. Molecular docking, molecular-dynamics simulations, and qPCR together strengthened the plausibility of an SHBG-associated rescue axis. Nevertheless, this should still be interpreted as a nominated mechanism. Direct target engagement, protein-level validation, tissue localization, and perturbation experiments demonstrating functional dependence on SHBG are still required before a direct-target claim can be justified.

Several limitations should be acknowledged. First, the human cohort was relatively small and age-unmatched, and the age ranges of the POR and control groups had limited overlap. Although age-adjusted sensitivity analyses were added, statistical adjustment cannot fully resolve this design limitation. Second, follicular fluid is a low-biomass specimen for 16S profiling. DNA extraction and PCR negative controls were included, but collection-site blank controls were not available; therefore, low-abundance microbial signals require cautious interpretation and future validation. Third, Harmalol and Beraprost were selected from MS/MS-annotated untargeted metabolomic features and were not confirmed by targeted LC-MS/MS. Fourth, the diagnostic models remain exploratory because they were developed in a single cohort without external validation. Fifth, the Tripterygium glycoside-induced ovarian injury model captures POR-like ovarian dysfunction but does not fully reproduce the clinical heterogeneity of human POR. Finally, although qPCR and SHBG immunohistochemistry support an SHBG-associated candidate mechanism, functional perturbation experiments are still required to establish causality. These limitations define the appropriate scope of interpretation.

Overall, our findings suggest that POR is associated with metabolic remodeling in FF, accompanied by exploratory microbial-signature changes and a recurrent steroidogenic response axis that may be partially reversible *in vivo*. The most valuable outcome of the present study is not a closed mechanistic conclusion, but a prioritized framework for future validation: metabolic remodeling as the more robust discovery layer, cautious microbiome-metabolome crosstalk as an exploratory layer, partial compound rescue as the translational layer, and an SHBG-associated steroidogenic program as a leading downstream hypothesis. Further age-controlled human studies, targeted metabolite confirmation, dedicated low-biomass microbiome validation, and direct experimental interrogation of SHBG and transport-related biology will be necessary to determine how far this framework can be translated into clinically actionable intervention strategies.

## 5 Conclusion

Integrated multi-omics analysis of follicular fluid identified metabolic remodeling and exploratory microbial-signature changes in poor ovarian response, with ABC transporter-related pathways emerging as a shared signal and steroidogenic dysregulation as a recurrent downstream theme. Candidate-compound interventions partially rescued endocrine and ovarian abnormalities in a mouse ovarian injury model, and ovarian transcriptomics together with qPCR and SHBG immunohistochemistry nominated an SHBG-associated steroidogenic program as a candidate mediator of this response. These findings provide a focused framework linking follicular microenvironment remodeling to testable rescue hypotheses in POR and define a bounded set of priorities for direct validation.

Integrated multi-omics analysis of follicular fluid identified coordinated microbial-signature and metabolic remodeling in poor ovarian response, with ABC transporter-related pathways emerging as the main shared signal and steroidogenic dysregulation as a recurrent downstream theme. Candidate-compound interventions partially rescued endocrine and ovarian abnormalities in a mouse ovarian injury model, and ovarian transcriptomics nominated an SHBG-associated steroidogenic program as a candidate mediator of this response. These findings provide a focused framework linking follicular microenvironment remodeling to testable rescue hypotheses in POR and define a bounded set of priorities for direct validation.

## Data Availability

The datasets presented in this study can be found in online repositories. The names of the repository/repositories and accession number(s) can be found below: https://www.ncbi.nlm.nih.gov/, PRJNA1335391.

## Supplementary material

Supplementary Figure 1 Analysis of differential metabolites between the POR and control groups. **(A)** Correlation network between differential microorganisms and metabolites. **(B)** Heatmap of differentially abundant metabolites between the POR and control groups. **(C)** Correlation network between differential microorganisms and metabolites.

Supplementary Figure 2 GO enrichment analysis of ovarian transcriptomes across different experimental groups. **(A)** Gene Ontology (GO) enrichment analysis of overlapping targets between Beraprost and POR. **(B)** GO enrichment analysis of overlapping targets between Harmalol and POR. **(C)** Molecular docking visualization of Beraprost with predicted core proteins. **(D)** Molecular docking visualization of Harmalol with predicted core proteins.

Supplementary Figure 3 Transcriptomic analysis of POR mouse models following endogenous metabolite interventions. **(A)** Representative images of vaginal smears and evaluation of estrous cycle phases. **(B)** KEGG pathway enrichment analysis of upregulated and downregulated DEGs in the POR (top left), M1 (top right), M2 (bottom left), and M3 (bottom right) groups. **(C)** GSEA of downregulated gene sets in the M1 (top left), M2 (top right), and M3 (bottom) groups. GSEA, Gene Set Enrichment Analysis; DEG, differentially expressed gene.
